# Structures of disodium hydrogen citrate mono­hydrate, Na_2_HC_6_H_5_O_7_(H_2_O), and di­ammonium sodium citrate, (NH_4_)_2_NaC_6_H_5_O_7_, from powder diffraction data

**DOI:** 10.1107/S2056989020011895

**Published:** 2020-09-04

**Authors:** Jerry Hong, Shivang Bhaskar, Joseph T. Golab, James A. Kaduk

**Affiliations:** a Illinois Mathematics and Science Academy, 1500 Sullivan Road, Aurora, IL 60506 , USA; b Department of Chemistry, North Central College, 131 S. Loomis, St., Naperville IL, 60540 , USA

**Keywords:** powder diffraction, citrate, sodium, ammonium, density functional theory

## Abstract

The crystal structures of disodium hydrogen citrate monohydrate, Na_2_HC_6_H_5_O_7_(H_2_O), and di­ammonium sodium citrate, (NH_4_)_2_NaC_6_H_5_O_7_, have been solved and refined using laboratory X-ray powder diffraction data, and optimized using density functional techniques. In NaHC_6_H_5_O_7_(H_2_O), the NaO_6_ coordination polyhedra share edges, forming zigzag layers lying parallel to the *bc* plane. The hydro­phobic methyl­ene groups occupy the inter­layer spaces. The carb­oxy­lic acid group makes a strong charge-assisted hydrogen bond to the central carboxyl­ate group. The hydroxyl group makes an intra­molecular hydrogen bond to an ionized terminal carboxyl­ate oxygen atom. Each hydrogen atom of the water mol­ecule acts as a donor, to a terminal carboxyl­ate and the hydroxyl group. Both the Na substructure and the hydrogen bonding differ from those of the known phase Na_2_HC_6_H_5_O_7_(H_2_O)_1.5_. In (NH_4_)_2_NaC_6_H_5_O_7_, the NaO_6_ coordination octa­hedra share corners, making double zigzag chains propagating along the *b*-axis direction. Each hydrogen atom of the ammonium ions acts as a donor in a discrete N—H⋯O hydrogen bond. The hydroxyl group forms an intra­molecular O—H⋯O hydrogen bond to a terminal carboxyl­ate oxygen atom.

## Abstract 2

The crystal structures of disodium hydrogen citrate monohydrate and di­ammonium sodium citrate have been solved and refined using laboratory X-ray powder diffraction data and optimized using density functional techniques.

## Chemical context   

A systematic study of the crystal structures of Group 1 (alkali metal) citrate salts has been reported in Rammohan & Kaduk (2018 ▸). The study was extended to ammonium citrates in Wheatley & Kaduk (2019 ▸). Na_2_HC_6_H_5_O_7_(H_2_O) was an accidental product of an extension of the program to mixed ammonium–group 1 citrates, and (NH_4_)_2_NaC_6_H_5_O_7_ was an intended product. Another product in the series is (NH_4_)_2_KC_6_H_5_O_7_ (Patel *et al.*, 2020 ▸). Known sodium citrates include two polymorphs of NaH_2_C_6_H_5_O_7_ (Rammohan & Kaduk, 2016*b* ▸; Glusker *et al.*, 1965 ▸), Na_2.5_H_0.5_C_6_H_5_O_7_ (Rammohan & Kaduk, 2017 ▸), Na_2_HC_6_H_5_O_7_(H_2_O)_1.5_ (Rammohan & Kaduk, 2016*c* ▸), Na_3_C_6_H_5_O_7_ (Rammohan & Kaduk, 2016*a* ▸), Na_3_C_6_H_5_O_7_(H_2_O)_2_ (Fischer & Palladino, 2003 ▸), and Na_3_C_6_H_5_O_7_(H_2_O)_5.5_ (Viossat *et al.*, 1986 ▸).

As part of our ongoing studies in this area, we now report the syntheses and structures of disodium hydrogen citrate monohydrate, Na_2_HC_6_H_5_O_7_(H_2_O), (I), and di­ammonium sodium citrate, (NH_4_)_2_NaC_6_H_5_O_7_, (II).
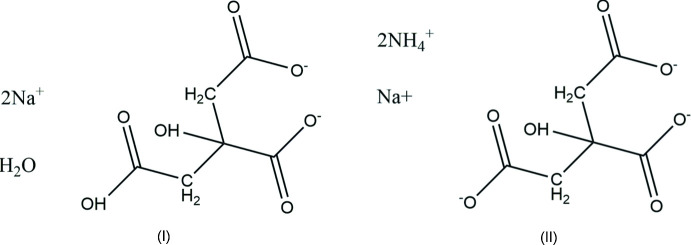


## Structural commentary   

**Figure 1:**
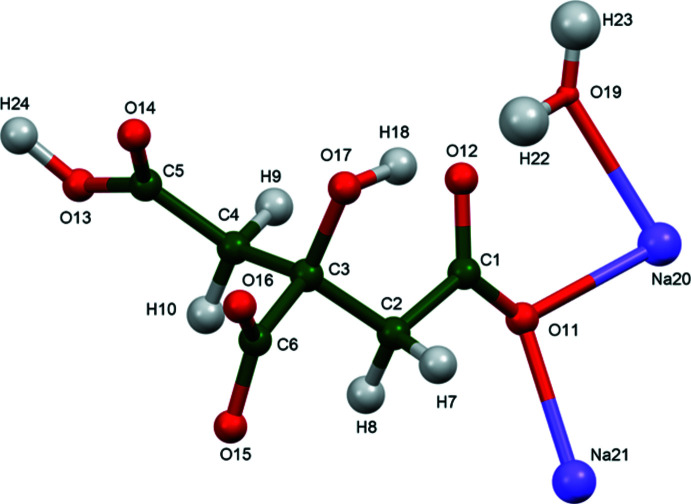
The asymmetric unit of (I) with the atom numbering and 50% probability spheres.

**Figure 2:**
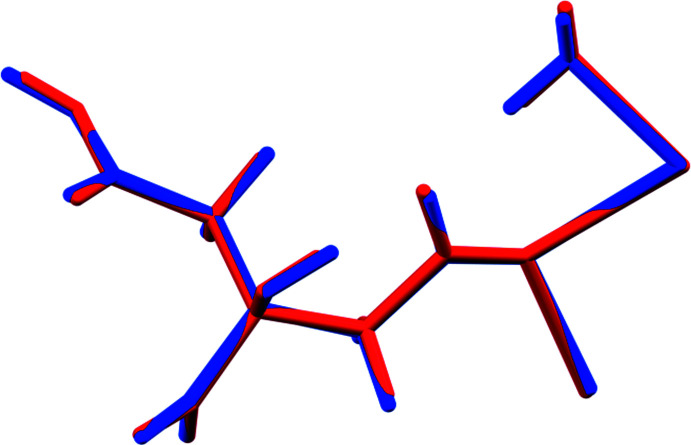
Comparison of the refined and optimized structures of (I): the refined structure is in red, and the DFT-optimized structure is in blue.

The structure of (I) was solved and refined from powder X-ray data and optimized by density functional theory (DFT) calculations (see *Experimental* section) and is illustrated in Fig. 1 ▸. The root-mean-square Cartesian displacement of the non-hydrogen citrate atoms in the Rietveld refined and DFT-optimized structures is 0.0764 Å (Fig. 2 ▸). The excellent agreement between the two structures is strong evidence that the experimental structure is correct (van de Streek & Neumann, 2014 ▸). All of the citrate bond distances, bond angles, and torsion angles fall within the normal ranges indicated by a *Mercury* Mogul geometry check (Macrae *et al.*, 2020 ▸). The citrate anion occurs in the *gauche, trans*-conformation (about C2—C3 and C3—C4, respectively), which is one of the two low-energy conformations of an isolated citrate ion (Rammohan & Kaduk, 2018 ▸). The central carboxyl­ate group and the hydroxyl group exhibit a small twist (O16—C6—C3—O17 torsion angle = 10.3°) from the normal planar arrangement. The Mulliken overlap populations indicate that the Na—O bonds are ionic. Both Na cations are six-coordinate (distorted octa­hedral). The bond-valence sums for Na20 and Na21 are 1.09 and 1.04 respectively.

The citrate anion triply chelates to Na20 through the terminal carboxyl­ate oxygen atom O14, the central carboxyl­ate oxygen atom O16, and the hydroxyl group O17. All oxygen atoms except O12 coordinate to at least one Na cation.

The Bravais–Friedel–Donnay–Harker (Bravais, 1866 ▸; Friedel, 1907 ▸; Donnay & Harker, 1937 ▸) method suggests that we might expect blocky morphology for disodium hydrogen citrate monohydrate. No preferred orientation model was necessary in the refinement.

**Figure 3:**
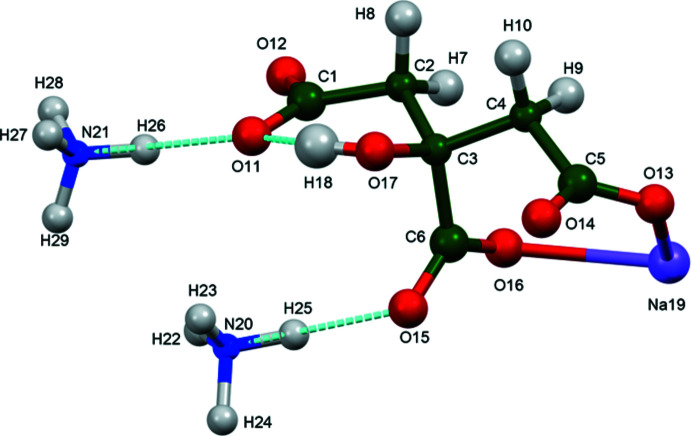
The asymmetric unit of (II) with the atom numbering and 50% probability spheres.

**Figure 4:**
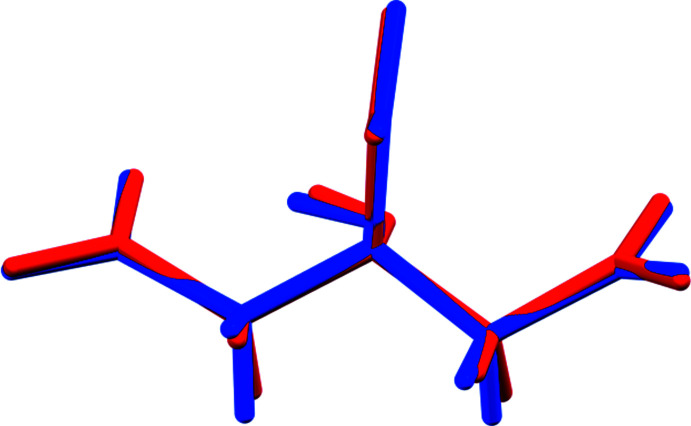
Comparison of the refined and optimized structures of (II): the refined structure is in red, and the DFT-optimized structure is in blue.

The structure of (II) was solved and refined from powder X-ray data and optimized by density functional theory (DFT) calculations (see *Experimental* section) and is illustrated in Fig. 3 ▸. The root-mean-square Cartesian displacement of the non-hydrogen citrate atoms in the Rietveld refined and DFT-optimized structures is 0.067 Å (Fig. 4 ▸). The r.m.s. displacement of the sodium ions is 0.037 Å and the equivalent values for the ammonium ions N20 and N21 are 0.148 and 0.147 Å, respectively. The excellent agreement between the two structures is strong evidence that the experimental structure is correct (van de Streek & Neumann, 2014 ▸). Almost all of the citrate bond distances, bond angles, and torsion angles fall within the normal ranges indicated by a *Mercury* Mogul geometry check (Macrae *et al.*, 2020 ▸). Only the O13—C5—C4 angle of 117.3° [average = 119.4 (7)°, 6-score = 3.2] is flagged as unusual. Mogul finds a population of three similar angles and the standard uncertainty is exceptionally low at 0.7°, so the *Z*-score is not of concern. The citrate anion occurs in the *trans, trans*-conformation (about C2—C3 and C3—C4), which is one of the two low-energy conformations of an isolated citrate ion (Rammohan & Kaduk, 2018 ▸). The central carboxyl­ate group and the hydroxyl group exhibit a very small twist [O17—C3—C6—O15 = 0.34°] from the normal planar arrangement. The Mulliken overlap populations indicate that the Na—O bonds are ionic.

The Bravais–Friedel–Donnay–Harker method suggests that we might expect platy morphology for di­ammonium sodium citrate, with {100} as the major faces. A 2nd order spherical harmonic model was included in the refinement. The texture index was only 1.006, indicating that preferred orientation was not significant in this rotated capillary specimen.

## Supra­molecular features   

**Figure 5:**
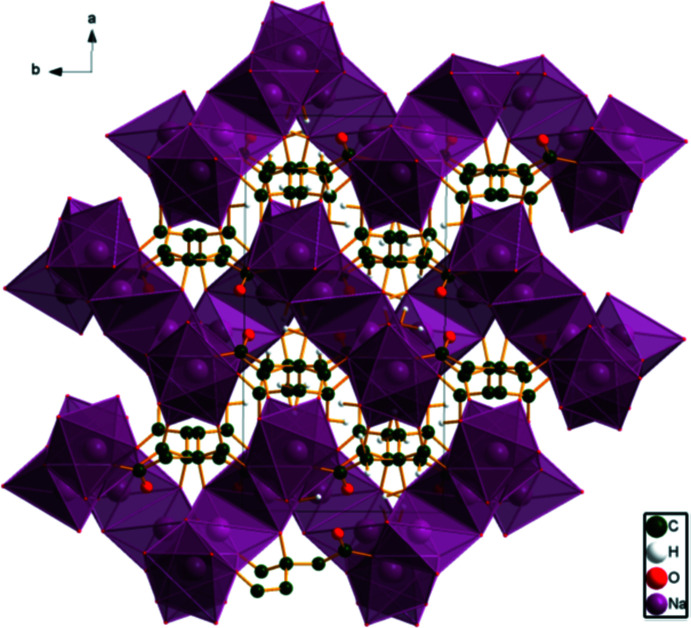
The crystal structure of (I) viewed down the *c* axis.

**Figure 6:**
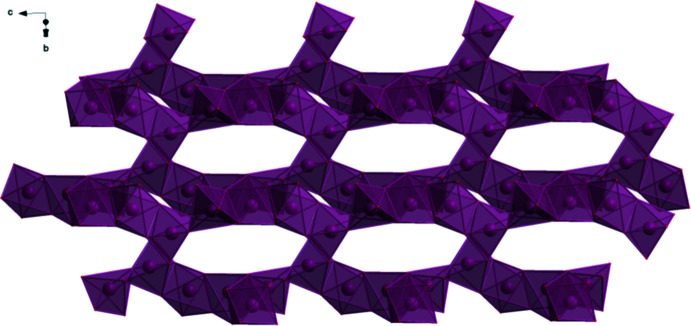
View of the Na/O layers in (I), viewed down [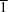
10].

In the extended structure of (I), the NaO_6_ coordination polyhedra share edges to form zigzag layers lying parallel to the *bc* plane (Fig. 5 ▸). The layers are conveniently viewed along [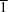
10] (Figs. 6 ▸ and 7 ▸). The hydro­phobic methyl­ene groups occupy the inter­layer spaces. The carb­oxy­lic acid O13—H24 group makes a strong (16.8 kcal mol^−1^) charge-assisted hydrogen bond to the central carboxyl­ate oxygen atom O15. The energies of the O—H⋯O hydrogen bonds were calculated using the correlation of Rammohan & Kaduk (2018 ▸). The hydroxyl group O17—H18 makes an intra­molecular hydrogen bond to the ionized terminal carboxyl­ate oxygen atom O12. Each hydrogen atom of the water mol­ecule O19 acts as a donor, to O12 and the hydroxyl group O17 (Table 1 ▸).

In the extended structure of Na_2_HC_6_H_5_O_7_(H_2_O)_1.5_ (Rammohan & Kaduk, 2016*c* ▸), the carb­oxy­lic acid makes a hydrogen bond to a terminal ionized carboxyl­ate group, while in this monohydrate, the –COOH group hydrogen bonds to the central ionized carboxyl­ate. In the sesquihydrate, the hydroxyl group hydrogen bonds to a terminal carboxyl­ate, while in this monohydrate the hydroxyl group forms an intra­molecular hydrogen bond. In the sesquihydrate, all three independent water mol­ecules bridge Na cations; in this monohydrate the water mol­ecule also bridges two Na. In the sesquihydrate, there are eight-membered rings of Na cations, while in this monohydrate structure the Na coordination spheres form layers.

The triclinic unit cell of Na_2_HC_6_H_5_O_7_(H_2_O)_1.5_ corresponds roughly to a 1/2 subcell of the current *Pbca* cell. The transformation matrix from the current cell to the standard ortho­rhom­bic cell is [0 1 0 / 0 0 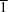 / 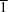 0 0], and the transformation matrix from the standard cell to the subcell is [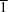 0 0 / −1/2 − 1/2 1/2 / −1/2 1/2 1/2]. Given the differences in the Na substructures and the hydrogen bonding, the similarities of the cells are a coincidence.

The *CRYSTAL14* (Dovesi *et al.*, 2014 ▸) energy per formula unit of Na_2_HC_6_H_5_O_7_(H_2_O) is −1160.0 eV. The energy per formula unit of Na_2_HC_6_H_5_O_7_(H_2_O)_1.5_ is −1197.9 eV. Calculated in the same way, the energy of an isolated water mol­ecule is −76.4 eV. Thus, the energy of the sesquihydrate is thus 0.23 eV higher than that of the sum of the monohydrate and half a water mol­ecule. The difference is only 5.4 kcal mol^−1^, so the structures must be considered comparable in energy.

**Figure 8:**
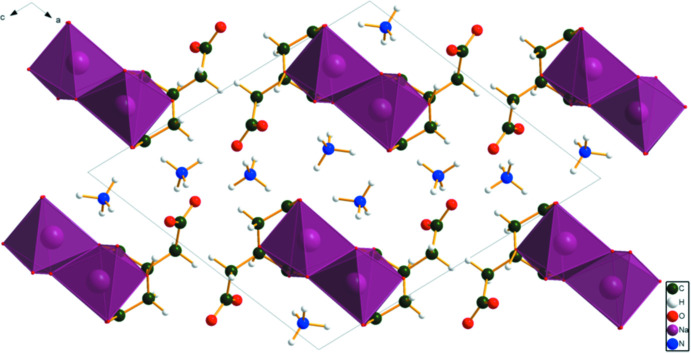
The crystal structure of (II), viewed down [010].

**Figure 10:**
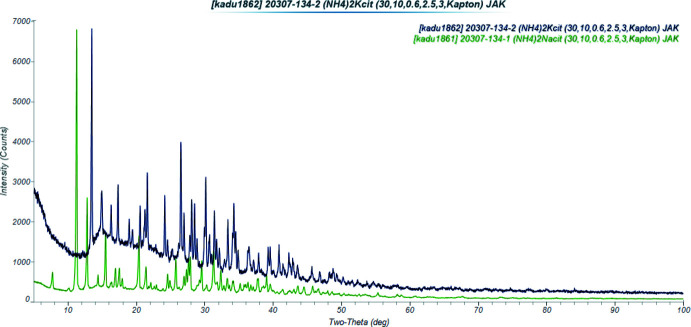
Comparison of the X-ray powder diffraction patterns of (II) (green) and (NH_4_)_2_KC_6_H_5_O_7_ (black).

**Figure 11:**
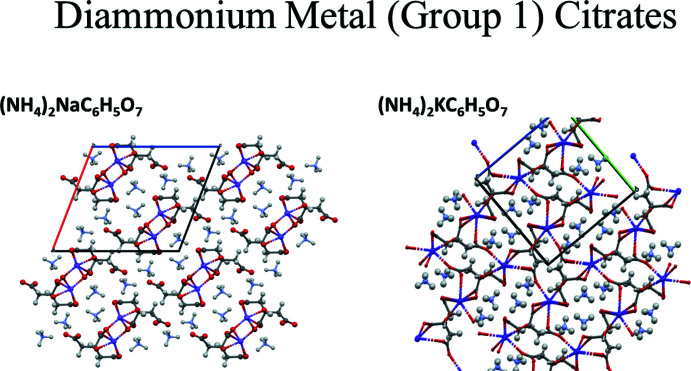
Comparison of the crystal structures of (II) and (NH_4_)_2_KC_6_H_5_O_7_.

In the extended structure of (II), the NaO_6_ coordination octa­hedra share corners to form double zigzag chains propagating along the *b*-axis direction (Figs. 8 ▸ and 9 ▸). Each hydrogen atom of the ammonium ions acts as a donor in a discrete N—H⋯O hydrogen bond (Table 2 ▸). The hydroxyl group O17—H18 forms an intra­molecular hydrogen bond to the terminal carboxyl­ate oxygen atom O11. The N—H⋯O hydrogen bond energies were calculated by the correlation of Wheatley & Kaduk (2019 ▸), and the O—H⋯O hydrogen bond energy was calculated by the correlation of Rammohan & Kaduk (2018 ▸). Despite the similarities in the formulae, the crystal structures of di­ammonium sodium citrate and di­ammonium potassium citrate (Patel *et al.*, 2020 ▸) differ. In the current compound, the NaO_6_ coordination polyhedra share corners to form zigzag chains, while in di­ammonium potassium citrate the KO_7_ polyhedra are isolated. The powder patterns (Fig. 10 ▸) are not particularly similar, and except for layers containing the ammonium ions (Fig. 11 ▸), the structures exhibit many differences.

## Database survey   

Details of the comprehensive literature search for citrate structures are presented in Rammohan & Kaduk (2018 ▸). Another pattern of the same sample of ‘(NH_4_)Na_2_C_6_H_5_O_7_’ measured using Cu *K*α radiation, was indexed on a primitive monoclinic unit cell having *a* = 16.9845, *b* = 8.6712, *c* = 12.2995 Å, *β* = 90.03°, *V* = 1800.2 Å^3^, and *Z* = 8 using *JADE Pro* (MDI, 2019 ▸). Analysis of the systematic absences using *FOX* (Favre-Nicolin & Černý, 2002 ▸) suggested that the space group was *Pbca*. A reduced cell search in the Cambridge Structural Database (Groom *et al.*, 2016 ▸) yielded 83 hits but no citrate crystal structures.

The pattern of (NH_4_)_2_NaC_6_H_5_O_7_ was indexed with *DICVOL14* (Louër & Boultif, 2014 ▸), using the *PreDICT* inter­face (Blanton *et al.*, 2019 ▸). Analysis of the systematic absences using *EXPO2014* (Altomare *et al.*, 2013 ▸) suggested the space group *P2_1_/n*, which was confirmed by successful solution and refinement of the structure. A reduced cell search of the cell in the Cambridge Structural Database (Groom *et al.*, 2016 ▸) resulted in eleven hits, but no citrate structures.

## Synthesis and crystallization   

0.2415 g of (NH_4_)_2_CO_3_ (Aldrich) and 0.5376 g of Na_2_CO_3_ (Alfa Aesar) were added to a solution of 1.0162 g citric acid (Sigma–Aldrich) monohydrate in 10 ml of water. After the fizzing subsided, the clear solution was dried at ambient conditions to yield a clear glass. Successive heating at 361, 394, and 410 K did not induce crystallization. The glass was redissolved in 10 ml of water and layered with 40 ml of ethanol. The beaker was covered and left to stand at ambient conditions. After three days, the solvents were blended, but the solution was clear. The beaker was uncovered and after another three days, a white solid was observed at the bottom of the beaker. The solution was deca­nted and the solid was dried at ambient conditions. After one day, the solid was still wet, so it was dried in a 361 K oven for a few minutes to yield a white powder of (I). The powder pattern was measured from a 0.7 mm diameter capillary specimen on a PANalytical Empyrean diffractometer equipped with an incident beam focusing mirror and an X’Celerator detector, using Mo *K*α radiation. The pattern was measured from 1–50° 2θ in 0.010067° steps, counting for four seconds per step.

Di­ammonium sodium citrate was synthesized by dissolving 1.1231 g di­ammonium hydrogen citrate (Fisher Lot #995047) and 0.2713 g sodium carbonate (Alfa Aesar) in ∼6 ml of deionized water. When the fizzing stopped, the clear solution was layered with about 20 ml of acetone and left to stand at ambient conditions. After two days, the solvents had blended and the product was a clear syrup. The syrup was dried at 363 K for three hours to yield a white solid, (II). The powder pattern was measured from a 0.7 mm diameter capillary specimen on a PANalytical Empyrean diffractometer equipped with an incident beam focusing mirror and an X’Celerator detector, using Mo *K*α radiation. The pattern was measured from 1–50° 2θ in 0.010067° steps, counting for four seconds per step.

## Refinement   

**Figure 12:**
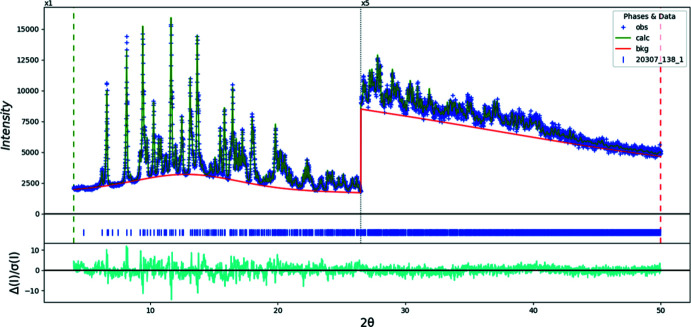
Rietveld plot for (I). The blue crosses represent the observed data points, and the green line is the calculated pattern. The cyan curve is the normalized error plot. The vertical scale has been multiplied by a factor of 5× for 2θ > 26.5°. The row of blue tick marks indicates the calculated reflection positions. The red line is the background curve.

Crystal data, data collection and structure refinement details for (I) and (II) are summarized in Table 3 ▸. The final Rietveld plots for (I) and (II) are shown in Figs. 12 ▸ and 13 ▸, respectively. The structure of (I) was solved using Monte-Carlo simulated annealing techniques as implemented in *FOX* (Favre-Nicolin & Černý 2002 ▸). The citrate anion, two sodium atoms and a nitro­gen atom were used as fragments. One of the fifteen runs yielded a cost factor much lower than the others and was used as the basis for refinement.

The structure was refined by the Rietveld method using *GSAS-II* (Toby & Von Dreele, 2013 ▸). In the initial refinement, the *U*_iso_ value of the nitro­gen atom refined to a negative value and the nitro­gen atom was 2.4 Å away from the two sodium atoms. Both of these facts suggested that this atom was not the nitro­gen of an ammonium ion, but the oxygen of a water mol­ecule. Thus, the compound was not the intended compound.

The hydrogen atoms were included in fixed positions, which were re-calculated during the course of the refinement using *Materials Studio* (Dassault Systems, 2019 ▸). Initial positions of the active hydrogen atoms H18, H22, H23, and H24 were deduced by analysis of potential hydrogen-bonding patterns. The *U*_iso_ values of C2, C3, and C4 were constrained to be equal, and those of H7, H8, H9, and H10 were constrained to be 1.3× that of these carbon atoms. The *U*_iso_ value of C1, C5, C6, and the oxygen atoms were constrained to be equal, and that of H18 was constrained to be 1.3× this value. The background was described by a four-term shifted Chebyshev polynomial with an extra peak at 12.85° to describe the scattering of the glass capillary.

A density functional geometry optimization was carried out using *CRYSTAL14* (Dovesi *et al.*, 2014 ▸). The basis sets for the H, C, N, and O atoms were those of Gatti *et al.* (1994 ▸), and the basis set for Na was that of Peintinger *et al.* (2013 ▸). The calculation was run on eight 2.1 GHz Xeon cores (each with 6 Gb RAM) of a 304-core Dell Linux cluster at IIT, using 8 *k*-points and the B3LYP functional, and took ∼44 h.

The structure of (II) was solved using *DASH* (David *et al.*, 2006 ▸) using a citrate ion, two nitro­gen atoms, and a sodium atom as fragments, along with Mogul Distribution Bias, and <010> preferred orientation. Two of the 100 runs yielded residuals lower than the others. The structure was refined by the Rietveld method using *GSAS-II* (Toby & Von Dreele, 2013 ▸). The hydrogen atoms were included in fixed positions, which were recalculated during the course of the refinement using *Materials Studio* (Dassault Systems, 2019 ▸). All C—C and C—O bond distances and all bond angles were restrained based on a *Mercury* Mogul Geometry Check (Sykes *et al.*, 2011 ▸; Bruno *et al.*, 2004 ▸) of the mol­ecule. The *U*_iso_ values of the atoms in the central and outer portions of the citrate were constrained to be equal, and the *U*_iso_ values of the hydrogen atoms were constrained to be 1.3× those of the atoms to which they are attached. A four-term shifted Chebyschev function was used to model the background, along with a peak at 12.5° to describe the scattering from the capillary and any amorphous component. A single phase model did not account for all of the peaks. We compared those peaks to the patterns of known ammonium and sodium citrates and identified di­ammonium hydrogen citrate (Wheatley & Kaduk, 2019 ▸) and disodium hydrogen citrate monohydrate [*i.e*., (I)] as impurities, and included them in the refinement. Their concentrations were 8.8% and 4.1% weight percentages respectively.

A density functional geometry optimization (fixed experimental unit cell) was carried out using *VASP* (Kresse & Furthmüller, 1996 ▸) through the *MedeA* graphical inter­face (Materials Design, 2016 ▸). The calculation was carried out on 16 2.4 GHz processors (each with 4 GB RAM) of a 64-processor HP Proliant DL580 Generation 7 Linux cluster at North Central College. The calculation used the GGA-PBE functional, a plane wave cutoff energy of 400.0 eV, and a *k*-point spacing of 0.5 Å^−1^ leading to a 2 × 3 × 2 mesh, and took 18 h. A single point calculation was done using *CRYSTAL14*. The basis sets for the H, C, N, and O atoms were those of Gatti *et al.* (1994 ▸), and the basis set for Na was that of Peintinger *et al.* (2013 ▸). The calculation was run on eight 2.1 GHz Xeon cores (each with 6 GB RAM) of a 304-core Dell Linux cluster at IIT, using 8 *k*-points and the B3LYP functional, and took five days.

## Supplementary Material

Crystal structure: contains datablock(s) global, I_DFT, kadu1875_publ, kadu1875_overall, II, II_imp1, kadu1875_pwd_0, II-imp2, I, II_DFT. DOI: 10.1107/S2056989020011895/hb7903sup1.cif

Supporting information file. DOI: 10.1107/S2056989020011895/hb7903II-imp2sup2.cml

CCDC references: 2025987, 2025986, 2025985, 2025984, 2025983, 2025982

Additional supporting information:  crystallographic information; 3D view; checkCIF report

## Figures and Tables

**Figure 7:**
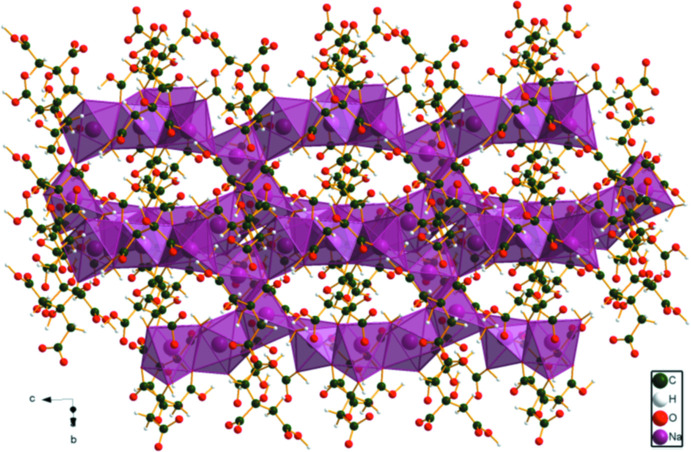
View of the crystal structure of (I), viewed down [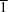
10].

**Figure 9:**
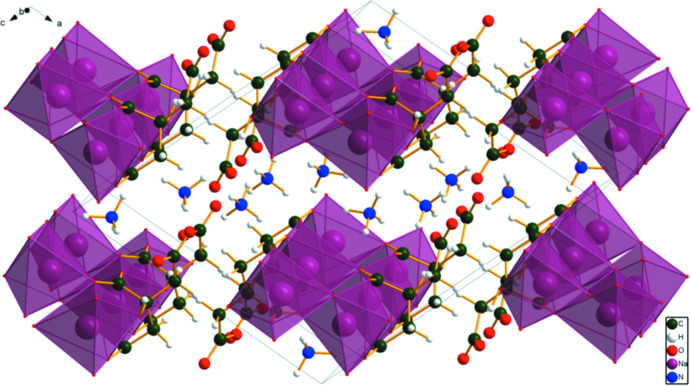
The crystal structure of (II), viewed nearly down the *b*-axis direction, to better illustrate the chains.

**Figure 13:**
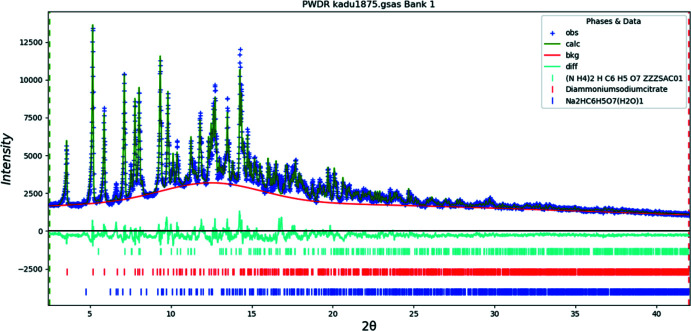
Rietveld plot for (II). The blue crosses represent the observed data points, and the green line is the calculated pattern. The cyan curve is the normalized error plot. The row of blue tick marks indicates the calculated reflection positions. The red and cyan tick marks indicate the reflection positions for the di­ammonium sodium citrate and di­ammonium hydrogen citrate impurities. The red line is the background curve.

**Table 1:** Hydrogen-bond geometry (Å, °) for (I) (DFT)

*D*—H⋯*A*	*D*—H	H⋯*A*	*D*⋯*A*	*D*—H⋯*A*
O13—H24⋯O15^i^	1.06	1.42	2.485	175
O17—H18⋯O12	1.00	1.62	2.578	179
O19—H23⋯O17^ii^	0.97	2.18	3.076	154
O19—H22⋯O12^iii^	1.00	1.62	2.615	172

Symmetry codes: (i) 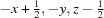; (ii) 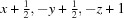; (iii) 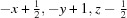.

**Table 2:** Hydrogen-bond geometry (Å, °) for (II) (DFT)

*D*—H⋯*A*	*D*—H	H⋯*A*	*D*⋯*A*	*D*—H⋯*A*
O17—H18⋯O11	0.99	1.75	2.630	146
N20—H22⋯O13^i^	1.04	1.76	2.798	177
N20—H23⋯O16^ii^	0.95	1.90	2.755	148
N20—H24⋯O14^i^	1.03	1.82	2.831	170
N20—H25⋯O15^iii^	1.03	1.82	2.831	167
N21—H26⋯O11	1.04	1.75	2.767	164
N21—H27⋯O12^iv^	1.04	1.69	2.730	176
N21—H28⋯O12^iii^	1.03	1.78	2.798	168
N21—H29⋯O14^v^	1.02	2.13	2.996	141

Symmetry codes: (i) 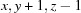; (ii) 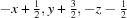; (iii) 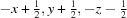; (iv) 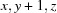; (v) 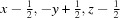.

**Table 3:** Experimental details

	(I)	(II)
Crystal data		
Chemical formula	2Na^+^·C_6_H_7_O_7_^2−^·H_2_O	C_6_H_13_N_2_NaO_7_
*M*_r_	254.1	248.17
Crystal system, space group	Orthorhombic, *P* *b* *c* *a*	Monoclinic, *P*2_1_/*n*
Temperature (K)	304	304
*a*, *b*, *c* (Å)	16.9976 (5), 8.6270 (2), 12.2926 (4)	13.0895 (19), 5.6403 (3), 14.822 (2)
α, β, γ (°)	90, 90, 90	90, 111.112 (4), 90
*V* (Å^3^)	1802.56 (12)	1020.83 (10)
*Z*	8	4
Radiation type	*K*α_1,2_, λ = 0.70932, 0.71361 Å	*K*α_1,2_, λ = 0.70932, 0.71361 Å
μ (mm^−1^)	0.086	0.104
Specimen shape, size (mm)	Cylinder, 12 × 0.7	Cylinder, 12 × 0.7

Data collection		
Diffractometer	PANalytical Empyrean	PANalytical Empyrean
Specimen mounting	Glass capillary	Glass capillary
Data collection mode	Transmission	Transmission
Scan method	Step	Step
2θ values (°)	2θ_min_ = 1.019 2θ_max_ = 49.999 2θ_step_ = 0.008	2θ_min_ = 1.019 2θ_max_ = 49.999 2θ_step_ = 0.008

Refinement		
*R* factors and goodness of fit	*R*_p_ = 0.031, *R*_wp_ = 0.040, *R*_exp_ = 0.020, *R*(*F* ^2^) = 0.06046, χ^2^ = 4.339	*R*_p_ = 0.046, *R*_wp_ = 0.061, *R*_exp_ = 0.020, χ^2^ = 9.703
No. of parameters	64	75
No. of restraints	–	29
H-atom treatment	Only H-atom displacement parameters refined	–

Computer programs: *FOX* (Favre-Nicolin & Černý, 2002 ▸), *GSAS-II* (Toby & Von Dreele, 2013 ▸), *Mercury* (Macrae *et al.*, 2020 ▸), *DIAMOND* (Crystal Impact, 2015 ▸), *publCIF* (Westrip, 2010 ▸).

## supplementary crystallographic information

### Disodium hydrogen citrate monohydrate (I) Crystal data

**Table:**

2Na^+^·C_6_H_7_O_7_^2^^−^·H_2_O	*D*_x_ = 1.873 Mg m^−^^3^
*M**_r_* = 254.1	*K*α_1,2_ radiation, λ = 0.70932, 0.71361 Å
Orthorhombic, *P**b**c**a*	*T* = 304 K
*a* = 16.9976 (5) Å	Particle morphology: white powder
*b* = 8.6270 (2) Å	white
*c* = 12.2926 (4) Å	cylinder, 12 × 0.7 mm
*V* = 1802.56 (12) Å^3^	Specimen preparation: Prepared at 298 K
*Z* = 8	

### Disodium hydrogen citrate monohydrate (I) Data collection

**Table:**

PANalytical Empyrean diffractometer	Data collection mode: transmission
Radiation source: sealed X-ray tube, PANalytical Empyrean	Scan method: step
Specimen mounting: glass capillary	2θ_min_ = 1.019°, 2θ_max_ = 49.999°, 2θ_step_ = 0.008°

### Disodium hydrogen citrate monohydrate (I) Refinement

**Table:**

Least-squares matrix: full	64 parameters
*R*_p_ = 0.031	Only H-atom displacement parameters refined
*R*_wp_ = 0.040	Weighting scheme based on measured s.u.'s
*R*_exp_ = 0.020	(Δ/σ)_max_ = 0.015
*R*(*F*^2^) = 0.06046	Background function: Background function: "chebyschev" function with 4 terms: 1682(4), -690(8), -285(6), 225(10), Background peak parameters: pos, int, sig, gam: 12.847, 1.463322627e6, 156931.773, 0.100,
5863 data points	Preferred orientation correction: March-Dollase correction coef. = 1.000 axis = [0, 0, 1]
Profile function: Finger-Cox-Jephcoat function parameters U, V, W, X, Y, SH/L: peak variance(Gauss) = Utan(Th)^2^+Vtan(Th)+W: peak HW(Lorentz) = X/cos(Th)+Ytan(Th); SH/L = S/L+H/L U, V, W in (centideg)^2^, X & Y in centideg 19.949, 12.795, 0.000, 2.075, 0.000, 0.032, Crystallite size in microns with "isotropic" model: parameters: Size, G/L mix 1.000, 1.000, Microstrain, "generalized" model (10^6^ * delta Q/Q) parameters: S400, S040, S004, S220, S202, S022, G/L mix 33.849, 17.475, 53.578, 71.795, -10.034, -20.359, 1.000,	

### Disodium hydrogen citrate monohydrate (I) Fractional atomic coordinates and isotropic or equivalent isotropic displacement parameters (Å^2^)

**Table:**

	*x*	*y*	*z*	*U*_iso_*/*U*_eq_	
C1	0.0915 (5)	0.5073 (6)	0.9674 (6)	0.0210 (9)*	
C2	0.1285 (4)	0.3821 (6)	1.0372 (4)	0.022 (2)*	
C3	0.1338 (3)	0.2175 (5)	0.9884 (4)	0.0216*	
C4	0.1952 (4)	0.2166 (6)	0.8963 (5)	0.0216*	
C5	0.2074 (4)	0.0741 (9)	0.8266 (6)	0.0210*	
C6	0.1579 (4)	0.1002 (7)	1.0781 (5)	0.0210*	
H7	0.10206	0.37794	1.10512	0.0280*	
H8	0.17906	0.41592	1.04805	0.0280*	
H9	0.18379	0.30361	0.84387	0.0280*	
H10	0.24619	0.24461	0.92993	0.0280*	
O11	0.1057 (3)	0.6434 (7)	0.9979 (5)	0.0210*	
O12	0.0560 (4)	0.4657 (7)	0.8835 (4)	0.0210*	
O13	0.2679 (4)	0.0826 (7)	0.7648 (5)	0.0210*	
O14	0.1527 (4)	−0.0242 (7)	0.8224 (4)	0.0210*	
O15	0.2199 (3)	0.1288 (7)	1.1304 (5)	0.0210*	
O16	0.1154 (3)	−0.0196 (6)	1.0820 (4)	0.0210*	
O17	0.0586 (3)	0.1802 (6)	0.9453 (4)	0.0210*	
O19	0.4752 (4)	0.2784 (7)	0.2712 (5)	0.010 (2)*	
Na20	0.0494 (2)	0.8835 (5)	0.9277 (3)	0.0371 (11)*	
Na21	0.6503 (3)	0.7676 (6)	0.8258 (3)	0.0371*	
H18	0.05760	0.27530	0.92410	0.0273*	
H22	0.46550	0.36160	0.30650	0.048*	
H23	0.49900	0.29090	0.20970	0.048*	
H24	0.27200	0.01000	0.71850	0.027*	

### Disodium hydrogen citrate monohydrate (I) Geometric parameters (Å, º)

**Table:**

Na20—O11	2.440 (8)	C4—C5	1.512 (2)
Na20—O14^i^	2.323 (6)	C4—H9	1.008 (7)
Na20—O16^i^	2.358 (6)	C4—H10	0.990 (7)
Na20—O17^i^	2.573 (6)	C5—C4	1.512 (2)
Na20—O17^ii^	2.470 (5)	C5—O13	1.280 (6)
Na20—O19^iii^	2.414 (7)	C5—O14	1.259 (6)
Na21—O11^iv^	2.421 (6)	C6—C3	1.553 (2)
Na21—O13^v^	2.392 (8)	C6—O15	1.258 (4)
Na21—O14^vi^	2.559 (7)	C6—O16	1.263 (5)
Na21—O15^vii^	2.441 (7)	H7—C2	0.949 (7)
Na21—O16^viii^	2.492 (6)	H7—H8	1.5208
Na21—O19^ix^	2.475 (6)	H8—C2	0.918 (7)
C1—C2	1.515 (2)	H8—H7	1.5208
C1—O11	1.256 (6)	H9—C4	1.008 (7)
C1—O12	1.248 (6)	H9—H10	1.5821
C2—C1	1.515 (2)	H10—C4	0.990 (7)
C2—C3	1.544 (2)	H10—H9	1.5821
C2—H7	0.949 (7)	O11—Na21^x^	2.421 (6)
C2—H8	0.918 (7)	O13—Na21^xi^	2.392 (8)
C3—C2	1.544 (2)	O13—H24	0.849 (5)
C3—C4	1.540 (2)	O17—H18	0.861 (5)
C3—C6	1.553 (2)	O19—H22	0.855 (6)
C3—O17	1.421 (2)	O19—H23	0.864 (6)
C4—C3	1.540 (2)		
			
C2—C1—O11	114.7 (4)	C5—C4—H9	105.6 (7)
C2—C1—O12	117.6 (4)	C3—C4—H10	106.5 (5)
O11—C1—O12	127.4 (4)	C5—C4—H10	108.4 (6)
C1—C2—C3	117.4 (3)	H9—C4—H10	104.7 (5)
C1—C2—H7	109.2 (6)	C4—C5—O13	113.5 (4)
C3—C2—H7	109.6 (5)	C4—C5—O14	118.0 (4)
C1—C2—H8	104.1 (6)	O13—C5—O14	127.4 (4)
C3—C2—H8	107.1 (5)	C3—C6—O15	117.2 (4)
H7—C2—H8	109.1 (5)	C3—C6—O16	114.2 (4)
C2—C3—C4	109.3 (4)	O15—C6—O16	128.3 (4)
C2—C3—C6	109.8 (4)	C5—O13—H24	115.0 (6)
C4—C3—C6	109.9 (4)	C5—O14—Na20^xii^	140.0 (5)
C2—C3—O17	107.5 (4)	C6—O16—Na20^xii^	122.2 (5)
C4—C3—O17	109.6 (4)	C3—O17—H18	85.0 (4)
C6—C3—O17	110.8 (3)	H22—O19—H23	115.4 (7)
C3—C4—C5	120.9 (4)	O14^i^—Na20—O16^i^	88.1 (2)
C3—C4—H9	109.6 (5)		

Symmetry codes: (i) *x*, *y*+1, *z*; (ii) −*x*, −*y*+1, −*z*+2; (iii) −*x*+1/2, −*y*+1, *z*+1/2; (iv) *x*+3/2, −*y*+5/2, −*z*+2; (v) −*x*+1, *y*+3/2, −*z*+5/2; (vi) *x*+3/2, *y*+1, −*z*+5/2; (vii) −*x*+1, −*y*+1, −*z*+2; (viii) *x*+3/2, −*y*+3/2, −*z*+2; (ix) −*x*+1, −*y*+1, −*z*+1; (x) *x*+1/2, −*y*+5/2, −*z*+2; (xi) −*x*+1, *y*+1/2, −*z*+5/2; (xii) *x*, *y*−1, *z*.

### (I_DFT) Crystal data

**Table:**

C6H8Na2O_8_	*b* = 8.6270 Å
*M**_r_* = 254.1	*c* = 12.2926 Å
Orthorhombic, *P**b**c**a*	*V* = 1808.6 Å^3^
*a* = 16.9976 Å	*Z* = 8

### (I_DFT) Data collection

**Table:**

*h* = →	*l* = →
*k* = →	

### (I_DFT) Fractional atomic coordinates and isotropic or equivalent isotropic displacement parameters (Å^2^)

**Table:**

	*x*	*y*	*z*	*U*_iso_*/*U*_eq_	
C1	0.09849	0.51407	0.97576	0.02100*	
C2	0.13266	0.38302	1.04450	0.02160*	
C3	0.13337	0.22227	0.98998	0.02160*	
C4	0.19515	0.22163	0.89890	0.02160*	
C5	0.20306	0.07952	0.82860	0.02100*	
C6	0.15362	0.09913	1.07587	0.02100*	
H7	0.09729	0.37727	1.11844	0.02800*	
H8	0.19189	0.41522	1.06874	0.02800*	
H9	0.18141	0.31609	0.84293	0.02800*	
H10	0.25308	0.24597	0.93282	0.02800*	
O11	0.11214	0.65091	1.00468	0.02100*	
O12	0.05644	0.47698	0.89371	0.02100*	
O13	0.26842	0.08179	0.77083	0.02100*	
O14	0.15525	−0.02732	0.82109	0.02100*	
O15	0.21561	0.12544	1.13214	0.02100*	
O16	0.11076	−0.01734	1.08635	0.02100*	
O17	0.05576	0.18923	0.94796	0.02100*	
H18	0.03863	0.29192	0.91925	0.02730*	
O19	0.47399	0.27963	0.27417	0.01010*	
Na20	0.04909	0.88130	0.92815	0.03710*	
Na21	0.64839	0.76211	0.82385	0.03710*	
H22	0.46390	0.37886	0.31416	0.04800*	
H23	0.51299	0.30171	0.21905	0.04800*	
H24	0.27350	−0.01033	0.71377	0.02700*	

### (I_DFT) Bond lengths (Å)

**Table:**

C1—C2	1.526	C4—H10	1.090
C1—O11	1.255	C5—O13	1.319
C1—O12	1.277	C5—O14	1.232
C2—C3	1.540	C6—O15	1.281
C2—H7	1.091	C6—O16	1.248
C2—H8	1.086	O13—H24	1.064
C3—C4	1.535	O17—H18	0.997
C3—C6	1.537	O19—H22	1.002
C3—O17	1.445	O19—H23	0.967
C4—C5	1.506	H24—O13	1.064
C4—H9	1.092		

### (I_DFT) Hydrogen-bond geometry (Å, º)

**Table:**

*D*—H···*A*	*D*—H	H···*A*	*D*···*A*	*D*—H···*A*
O13—H24···O15^i^	1.06	1.42	2.485	175
O17—H18···O12	1.00	1.62	2.578	179
O19—H23···O17^ii^	0.97	2.18	3.076	154
O19—H22···O12^iii^	1.00	1.62	2.615	172

Symmetry codes: (i) −*x*+1/2, −*y*, *z*−1/2; (ii) *x*+1/2, −*y*+1/2, −*z*+1; (iii) −*x*+1/2, −*y*+1, *z*−1/2.

### (II) Crystal data

**Table:**

C_6_H_13_N_2_NaO_7_	β = 111.112 (4)°
*M**_r_* = 248.17	*V* = 1020.83 (10) Å^3^
Monoclinic, *P*2_1_/*n*	*Z* = 4
*a* = 13.0895 (19) Å	*D*_x_ = 1.615 Mg m^−^^3^
*b* = 5.6403 (3) Å	*T* = 304 K
*c* = 14.822 (2) Å	cylinder, 12 × 0.7 mm

### (II) Data collection

**Table:**

PANalytical Empyrean diffractometer	Data collection mode: transmission
Specimen mounting: glass capillary	Scan method: step

### (II) Refinement

**Table:**

Profile function: Crystallite size in microns with "isotropic" model: parameters: Size, G/L mix 3.7(16), 1.000, Microstrain, "isotropic" model (10^6^ * delta Q/Q) parameters: Mustrain, G/L mix 100.000, 1.000,	Preferred orientation correction: Simple spherical harmonic correction Order = 2 Coefficients: 0:0:C(2,-2) = 0.0720; 0:0:C(2,0) = 0.0861; 0:0:C(2,2) = -0.0293
29 restraints	

### (II) Fractional atomic coordinates and isotropic or equivalent isotropic displacement parameters (Å^2^)

**Table:**

	*x*	*y*	*z*	*U*_iso_*/*U*_eq_	
C1	0.3245 (9)	−0.1347 (13)	−0.0540 (4)	0.0376 (19)*	
C2	0.4091 (8)	−0.2749 (16)	0.0265 (4)	0.025 (5)*	
C3	0.4171 (4)	−0.2134 (12)	0.1306 (4)	0.025*	
C4	0.5123 (6)	−0.3478 (19)	0.2064 (4)	0.025*	
C5	0.5063 (11)	−0.3558 (15)	0.3071 (5)	0.0376*	
C6	0.3075 (6)	−0.2837 (13)	0.1436 (9)	0.0376*	
H7	0.39850	−0.42995	0.01772	0.032*	
H8	0.47667	−0.21910	0.02837	0.032*	
H9	0.51195	−0.49468	0.18844	0.032*	
H10	0.57551	−0.24872	0.21107	0.032*	
O11	0.3120 (8)	0.0798 (12)	−0.0330 (6)	0.0376*	
O12	0.2890 (9)	−0.2373 (16)	−0.1363 (5)	0.0376*	
O13	0.5086 (9)	−0.5624 (16)	0.3416 (6)	0.0376*	
O14	0.5043 (9)	−0.1556 (18)	0.3462 (6)	0.0376*	
O15	0.2566 (7)	−0.1144 (17)	0.1649 (8)	0.0376*	
O16	0.2843 (7)	−0.5035 (14)	0.1339 (9)	0.0376*	
O17	0.4375 (7)	0.0334 (13)	0.1467 (7)	0.0376*	
H18	0.40000	0.10000	0.10000	0.0489*	
Na19	0.6357 (6)	−0.1785 (14)	0.7351 (6)	0.033 (3)*	
N20	0.3773 (10)	0.727 (2)	−0.5343 (9)	0.025 (5)*	
N21	0.1886 (10)	0.361 (3)	−0.1861 (9)	0.025*	
H22	0.42431	0.64858	0.53506	0.033*	
H23	0.33915	0.83022	0.45187	0.033*	
H24	0.44313	0.75229	0.42362	0.033*	
H25	0.34392	0.55514	0.42310	0.033*	
H26	0.22251	0.23759	0.86521	0.033*	
H27	0.22667	0.52482	0.83677	0.033*	
H28	0.17884	0.32689	0.74675	0.033*	
H29	0.10441	0.39477	0.81568	0.033*	

### (II) Geometric parameters (Å, º)

**Table:**

C1—C2	1.524 (3)	O16—C6	1.272 (3)
C1—O11	1.275 (3)	O16—Na19^iii^	2.572 (12)
C1—O12	1.277 (3)	O17—C3	1.422 (3)
C2—C1	1.524 (3)	O17—H18	0.785 (10)
C2—C3	1.549 (3)	O17—Na19^iv^	2.422 (10)
C2—H7	0.887 (10)	H18—O17	0.785 (10)
C2—H8	0.930 (11)	Na19—O13^iii^	2.335 (12)
C3—C2	1.549 (3)	Na19—O14^iv^	2.602 (15)
C3—C4	1.543 (3)	Na19—O15^iv^	2.325 (11)
C3—C6	1.565 (3)	Na19—O15^vi^	2.477 (11)
C3—O17	1.422 (3)	Na19—O16^iii^	2.572 (12)
C3—H24^i^	1.189 (5)	Na19—O17^iv^	2.422 (10)
C4—C3	1.543 (3)	N20—H22^ii^	0.881 (13)
C4—C5	1.524 (3)	N20—H23^ii^	0.963 (13)
C4—H9	0.869 (10)	N20—H25^ii^	1.077 (12)
C4—H10	0.980 (10)	N20—H29^vii^	1.133 (12)
C5—C4	1.524 (3)	N21—H26^ii^	1.015 (12)
C5—O13	1.268 (3)	N21—H27^ii^	1.107 (14)
C5—O14	1.274 (3)	N21—H28^ii^	1.104 (13)
C6—C3	1.565 (3)	H22—N20^viii^	0.881 (13)
C6—O15	1.268 (3)	H22—H23	1.6507
C6—O16	1.272 (3)	H22—H25	1.5431
C6—H24^i^	0.678 (7)	H22—H29^ix^	1.1533
H7—C2	0.887 (10)	H23—N20^viii^	0.963 (13)
H7—H8	1.5394	H23—H22	1.6507
H8—C2	0.930 (11)	H23—H25	1.3917
H8—H7	1.5394	H23—H29^ix^	1.0358
H9—C4	0.869 (10)	H24—C3^x^	1.189 (5)
H9—H10	1.5900	H24—C6^x^	0.678 (7)
H10—C4	0.980 (10)	H24—O15^x^	1.262 (5)
H10—H9	1.5900	H25—N20^viii^	1.077 (12)
O11—C1	1.275 (3)	H25—H22	1.5431
O11—H27^ii^	1.666 (7)	H25—H23	1.3917
O12—C1	1.277 (3)	H26—N21^viii^	1.015 (12)
O13—C5	1.268 (3)	H27—O11^viii^	1.666 (7)
O13—Na19^iii^	2.335 (12)	H27—N21^viii^	1.107 (14)
O14—C5	1.274 (3)	H27—H28	1.2772
O14—Na19^iv^	2.602 (15)	H28—N21^viii^	1.104 (13)
O15—C6	1.268 (3)	H28—H27	1.2772
O15—Na19^iv^	2.325 (11)	H29—N20^xi^	1.133 (12)
O15—Na19^v^	2.477 (11)	H29—H22^xii^	1.1533
O15—H24^i^	1.262 (5)	H29—H23^xii^	1.0358
			
C2—C1—O11	115.0 (3)	O16—C6—H24^i^	152.5 (12)
C2—C1—O12	115.1 (3)	C5—O13—Na19^iii^	118.8 (10)
O11—C1—O12	129.0 (3)	C6—O15—Na19^iv^	116.2 (6)
C1—C2—C3	115.7 (3)	C6—O15—H24^i^	31.1 (3)
C1—C2—H7	111.5 (7)	Na19^iv^—O15—H24^i^	96.1 (5)
C3—C2—H7	108.5 (6)	C3—O17—H18	107.5 (7)
C1—C2—H8	105.2 (8)	O13^iii^—Na19—O15^iv^	162.5 (5)
C3—C2—H8	99.9 (6)	H22^ii^—N20—H23^ii^	127.1 (13)
H7—C2—H8	115.7 (6)	H22^ii^—N20—H25^ii^	103.6 (13)
C2—C3—C4	111.2 (3)	H23^ii^—N20—H25^ii^	85.9 (10)
C2—C3—O17	109.1 (3)	H22^ii^—N20—H29^vii^	68.6 (8)
C4—C3—O17	107.8 (3)	H23^ii^—N20—H29^vii^	58.6 (7)
C2—C3—H24^i^	109.1 (4)	H25^ii^—N20—H29^vii^	98.2 (10)
C4—C3—H24^i^	127.4 (4)	H26^ii^—N21—H27^ii^	108.3 (13)
O17—C3—H24^i^	89.1 (4)	H26^ii^—N21—H28^ii^	106.3 (13)
C3—C4—C5	114.5 (3)	H27^ii^—N21—H28^ii^	70.6 (9)
C3—C4—H9	109.4 (6)	N20^viii^—H22—H29^ix^	66.1 (8)
C5—C4—H9	106.0 (6)	N20^viii^—H23—H29^ix^	69.0 (7)
C3—C4—H10	102.2 (6)	C3^x^—H24—C6^x^	110.9 (7)
C5—C4—H10	106.7 (7)	C3^x^—H24—O15^x^	156.2 (6)
H9—C4—H10	118.4 (7)	C6^x^—H24—O15^x^	75.0 (4)
C4—C5—O13	114.8 (3)	N21^viii^—H27—H28	54.6 (7)
C4—C5—O14	115.9 (3)	N21^viii^—H28—H27	54.8 (7)
O13—C5—O14	129.2 (3)	N20^xi^—H29—H22^xii^	45.3 (7)
O15—C6—O16	129.5 (3)	N20^xi^—H29—H23^xii^	52.5 (7)
O15—C6—H24^i^	74.0 (5)	H22^xii^—H29—H23^xii^	97.74

Symmetry codes: (i) *x*, *y*−1, *z*; (ii) *x*, *y*, *z*−1; (iii) −*x*+1, −*y*−1, −*z*+1; (iv) −*x*+1, −*y*, −*z*+1; (v) *x*+1/2, −*y*+1/2, *z*+1/2; (vi) *x*+3/2, −*y*+1/2, *z*+3/2; (vii) −*x*+1/2, *y*+1/2, −*z*−1/2; (viii) *x*, *y*, *z*+1; (ix) −*x*+1/2, *y*+1/2, −*z*+1/2; (x) *x*, *y*+1, *z*; (xi) −*x*+1/2, *y*−1/2, −*z*−1/2; (xii) −*x*+1/2, *y*−1/2, −*z*+1/2.

### (II_imp1) Crystal data

**Table:**

C_6_H_8_Na_2_O_8_	*c* = 12.304 (9) Å
*M**_r_* = 254.1	*V* = 1808.2 (16) Å^3^
Orthorhombic, *P**b**c**a*	*Z* = 8
*a* = 17.204 (13) Å	*D*_x_ = 1.867 Mg m^−^^3^
*b* = 8.542 (7) Å	*T* = 304 K

### (II_imp1) Refinement

**Table:**

Profile function: Crystallite size in microns with "isotropic" model: parameters: Size, G/L mix 1.000, 1.000, Microstrain, "isotropic" model (10^6^ * delta Q/Q) parameters: Mustrain, G/L mix 1000.000, 1.000,	Preferred orientation correction: March-Dollase correction coef. = 1.000 axis = [0, 0, 1]

### (II_imp1) Fractional atomic coordinates and isotropic or equivalent isotropic displacement parameters (Å^2^)

**Table:**

	*x*	*y*	*z*	*U*_iso_*/*U*_eq_	
C1	0.20530	0.07620	0.16930	0.018*	
C2	0.19420	0.22000	0.09980	0.018*	
C3	0.13112	0.21770	0.01070	0.018*	
C4	0.12960	0.37930	−0.04170	0.018*	
C5	0.09300	0.50710	0.02830	0.018*	
C6	0.15710	0.09790	−0.07630	0.018*	
H7	0.24819	0.25822	0.07204	0.024*	
H8	0.18236	0.30440	0.15185	0.024*	
H9	0.17863	0.41770	−0.05810	0.024*	
H10	0.09479	0.37506	−0.09986	0.024*	
O11	0.15210	−0.02310	0.17960	0.018*	
O12	0.26510	0.08280	0.22980	0.018*	
O13	0.10650	0.64490	0.00320	0.018*	
O14	0.05770	0.46920	0.11270	0.018*	
O15	0.11604	−0.02130	−0.08380	0.018*	
O16	0.21778	0.12760	−0.12890	0.018*	
O17	0.05818	0.17660	0.05080	0.018*	
H18	0.05800	0.27050	0.07740	0.024*	
O19	0.02810	0.72330	0.22740	0.025*	
Na20	0.65196	0.73330	0.67230	0.032*	
Na21	0.95010	0.11740	0.92864	0.032*	
H22	0.03370	0.63860	0.19410	0.039*	
H23	0.00030	0.70780	0.29040	0.039*	
H24	0.27170	0.00820	0.27920	0.025*	

### (II_imp1) Geometric parameters (Å, º)

**Table:**

C1—C2	1.5088	O14—H18	1.752
C1—O11	1.2543	O15—C6	1.2427
C1—O12	1.2711	O15—Na20^v^	2.4417
C2—C1	1.5088	O15—Na21^ii^	2.3693
C2—C3	1.5427	O16—C6	1.2542
C2—H7	1.0421	O16—Na20^vi^	2.4743
C2—H8	0.9856	O16—H24^vii^	1.6300
C3—C2	1.5427	O17—C3	1.3933
C3—C4	1.5238	O17—H18	0.8663
C3—C6	1.5469	O17—Na21^viii^	2.4438
C3—O17	1.3933	O17—Na21^ii^	2.5281
C3—H18	1.5683	H18—C3	1.5683
C4—C3	1.5238	H18—O14	1.752
C4—C5	1.5265	H18—O17	0.8663
C4—H9	0.9273	O19—Na20^ix^	2.4902
C4—H10	0.9339	O19—Na21^iii^	2.3830
C5—C4	1.5265	O19—H22	0.8370
C5—O13	1.2389	O19—H23	0.9204
C5—O14	1.2458	Na20—O11^x^	2.5583
C6—C3	1.5469	Na20—O12^iii^	2.4402
C6—O15	1.2427	Na20—O13^xi^	2.4176
C6—O16	1.2542	Na20—O15^xii^	2.4417
H7—C2	1.0421	Na20—O16^xiii^	2.4743
H7—H8	1.5500	Na20—O19^xiv^	2.4902
H8—C2	0.9856	Na21—O11^ii^	2.3482
H8—H7	1.5500	Na21—O13^iii^	2.4030
H9—C4	0.9273	Na21—O15^ii^	2.3693
H9—H10	1.5739	Na21—O17^xv^	2.4438
H10—C4	0.9339	Na21—O17^ii^	2.5281
H10—H9	1.5739	Na21—O19^iii^	2.3830
O11—C1	1.2543	Na21—Na21^xvi^	3.1709
O11—Na20^i^	2.5583	Na21—H22^iii^	2.5890
O11—Na21^ii^	2.3482	H22—O19	0.8370
O12—C1	1.2711	H22—Na21^iii^	2.5890
O12—Na20^iii^	2.4402	H22—H23	1.4435
O12—H24	0.8879	H23—O19	0.9204
O13—C5	1.2389	H23—H22	1.4435
O13—Na20^iv^	2.4176	H24—O12	0.8879
O13—Na21^iii^	2.4030	H24—O16^xvii^	1.6300
O14—C5	1.2458		
			
C2—C1—O11	121.028	C3—C4—H10	107.465
C2—C1—O12	113.469	C5—C4—H10	101.28
O11—C1—O12	124.153	H9—C4—H10	115.487
C1—C2—C3	118.779	C4—C5—O13	117.484
C1—C2—H7	109.148	C4—C5—O14	119.051
C3—C2—H7	113.456	O13—C5—O14	123.099
C1—C2—H8	104.677	C3—C6—O15	115.416
C3—C2—H8	109.009	C3—C6—O16	117.631
H7—C2—H8	99.673	O15—C6—O16	126.91
C2—C3—C4	107.531	C1—O11—Na21^ii^	136.141
C2—C3—C6	107.275	C1—O12—H24	118.201
C4—C3—C6	108.146	C6—O15—Na21^ii^	119.805
C2—C3—O17	112.649	C3—O17—H18	84.475
C4—C3—O17	111.263	H22—O19—H23	110.349
C6—C3—O17	109.791	O11^ii^—Na21—O15^ii^	88.773
C3—C4—C5	114.595	O11^ii^—Na21—Na21^xvi^	120.17
C3—C4—H9	113.387	O15^ii^—Na21—Na21^xvi^	66.085
C5—C4—H9	104.184		

Symmetry codes: (i) *x*+1/2, −*y*+3/2, −*z*+1; (ii) −*x*+1, −*y*, −*z*+1; (iii) −*x*+1, −*y*+1, −*z*+1; (iv) *x*+1/2, *y*, −*z*+3/2; (v) *x*+1/2, *y*−1, −*z*+3/2; (vi) −*x*+1, *y*+1/2, −*z*+3/2; (vii) −*x*+1/2, −*y*, *z*−1/2; (viii) *x*−1, *y*, *z*−1; (ix) *x*+1/2, −*y*+5/2, −*z*+1; (x) *x*+3/2, −*y*+3/2, −*z*+1; (xi) *x*+3/2, *y*, −*z*+3/2; (xii) *x*+3/2, *y*+1, −*z*+3/2; (xiii) −*x*+1, *y*+3/2, −*z*+3/2; (xiv) *x*+3/2, −*y*+5/2, −*z*+1; (xv) *x*+1, *y*, *z*+1; (xvi) −*x*+2, −*y*, −*z*+2; (xvii) −*x*+1/2, −*y*, *z*+1/2.

### Diammonium sodium citrate (II-imp2) Crystal data

**Table:**

NH_4_^+^·2Na^+^·C_6_H_5_O_7_^3^^−^	*V* = 983.0 (3) Å^3^
*M**_r_* = 227.19	*Z* = 4
Orthorhombic, *P**n**m**a*	*D*_x_ = 1.535 Mg m^−^^3^
*a* = 10.789 (3) Å	*T* = 304 K
*b* = 14.761 (4) Å	Particle morphology: white powder
*c* = 6.1723 (19) Å	

### Diammonium sodium citrate (II-imp2) Refinement

**Table:**

Profile function: Crystallite size in microns with "isotropic" model: parameters: Size, G/L mix 10(11), 1.000, Microstrain, "isotropic" model (10^6^ * delta Q/Q) parameters: Mustrain, G/L mix 1000.000, 1.000,	Preferred orientation correction: March-Dollase correction coef. = 1.000 axis = [0, 0, 1]

### Diammonium sodium citrate (II-imp2) Fractional atomic coordinates and isotropic or equivalent isotropic displacement parameters (Å^2^)

**Table:**

	*x*	*y*	*z*	*U*_iso_*/*U*_eq_	
O1	0.55311	0.05170	0.12400	0.030*	
O2	0.39069	0.04291	0.34322	0.030*	
O3	0.36355	0.25000	−0.03610	0.030*	
O4	0.57135	0.25000	−0.04560	0.030*	
O5	0.34625	0.25000	0.37810	0.030*	
H1	0.30500	0.25000	0.26300	0.039*	
C1	0.48748	0.07947	0.28340	0.030*	
C2	0.53601	0.16454	0.39030	0.030*	
H2	0.62560	0.17080	0.36200	0.039*	
H3	0.52040	0.16290	0.54200	0.039*	
C3	0.46985	0.25000	0.04960	0.030*	
C4	0.47041	0.25000	0.30190	0.030*	
N1	0.24446	0.11395	0.68150	0.030*	
H4	0.20300	0.06820	0.74600	0.039*	
H5	0.18200	0.15080	0.62400	0.039*	
H6	0.29200	0.14680	0.78600	0.039*	
H7	0.29800	0.09420	0.56700	0.039*	
H8?	0.50300	0.01500	0.03000	0.039*	

### Diammonium sodium citrate (II-imp2) Geometric parameters (Å, º)

**Table:**

O1—C1	1.2796	C4—C2^ii^	1.5460
O1—H8?	0.9604	C4—C3	1.5573
O1—H8?^i^	1.4965	N1—H4	0.9026
O2—C1	1.2321	N1—H5	0.9359
O3—C3	1.2630	N1—H6	0.9562
O4—C3	1.2428	N1—H7	0.9582
O5—H1	0.8383	H4—N1	0.9026
O5—C4	1.4198	H4—H5	1.4509
H1—O5	0.8383	H4—H6	1.5262
C1—O1	1.2796	H4—H7	1.5552
C1—O2	1.2321	H5—N1	0.9359
C1—C2	1.5121	H5—H4	1.4509
C2—C1	1.5121	H5—H6	1.5530
C2—H2	0.9866	H5—H7	1.5454
C2—H3	0.9517	H6—N1	0.9562
C2—C4	1.5460	H6—H4	1.5262
H2—C2	0.9866	H6—H5	1.5530
H2—H3	1.5926	H6—H7	1.5602
H3—C2	0.9517	H7—N1	0.9582
H3—H2	1.5926	H7—H4	1.5552
C3—O3	1.2630	H7—H5	1.5454
C3—O4	1.2428	H7—H6	1.5602
C3—C4	1.5573	H8?—O1	0.9604
C4—O5	1.4198	H8?—O1^i^	1.4965
C4—C2	1.5460	H8?—H8?^i^	0.5809
			
C1—O1—H8?	109.49	O5—C4—C2	108.365
H1—O5—C4	102.72	O5—C4—C2^ii^	108.365
O1—C1—O2	123.995	C2—C4—C2^ii^	109.369
O1—C1—C2	114.199	O5—C4—C3	109.123
O2—C1—C2	121.768	C2—C4—C3	110.776
C1—C2—H2	109.864	C2^ii^—C4—C3	110.776
C1—C2—H3	110.299	H4—N1—H5	104.196
H2—C2—H3	110.485	H4—N1—H6	110.351
C1—C2—C4	111.413	H5—N1—H6	110.324
H2—C2—C4	108.038	H4—N1—H7	113.358
H3—C2—C4	106.677	H5—N1—H7	109.343
O3—C3—O4	127.025	H6—N1—H7	109.173
O3—C3—C4	114.982	O1—H8?—H8?^i^	151.385
O4—C3—C4	117.994		

Symmetry codes: (i) −*x*+1, −*y*, −*z*; (ii) *x*, −*y*+1/2, *z*.

### (II_DFT) Crystal data

**Table:**

C_6_H_13_N_2_NaO_7_	*c* = 14.809688 Å
*M**_r_* = 248.17	β = 111.1167°
Monoclinic, *P*2_1_/*n*	*V* = 1018.21 Å^3^
*a* = 13.077726 Å	*Z* = 4
*b* = 5.635725 Å	

### (II_DFT) Data collection

**Table:**

*h* = →	*l* = →
*k* = →	

### (II_DFT) Fractional atomic coordinates and isotropic or equivalent isotropic displacement parameters (Å^2^)

**Table:**

	*x*	*y*	*z*	*U*_iso_*/*U*_eq_	
C1	0.334955	−0.125859	−0.053293	0.0368	
C2	0.413701	−0.273363	0.028415	0.025	
C3	0.417837	−0.212340	0.130913	0.025	
C4	0.513660	−0.347223	0.205316	0.025	
C5	0.510260	−0.352184	0.307173	0.0368	
C6	0.309818	−0.292988	0.142607	0.0368	
H7	0.394012	−0.460032	0.013463	0.032	
H8	0.495328	−0.244332	0.025980	0.032	
H9	0.514380	−0.528620	0.180811	0.032	
H10	0.589251	−0.261937	0.207408	0.032	
O11	0.312585	0.083690	−0.035269	0.0368	
O12	0.297573	−0.217642	−0.136819	0.0368	
O13	0.508939	−0.554338	0.344614	0.0368	
O14	0.506494	−0.157356	0.348963	0.0368	
O15	0.251139	−0.141227	0.164651	0.0368	
O16	0.288573	−0.511281	0.131537	0.0368	
O17	0.436659	0.036963	0.148371	0.0368	
H18	0.398865	0.115538	0.088678	0.048	
Na19	0.636591	−0.180023	0.738832	0.043	
N20	0.386650	0.697590	−0.536966	0.018	
H22	0.424318	0.648583	−0.464944	0.023	
H23	0.339152	0.830221	−0.548135	0.023	
H24	0.443135	0.752293	−0.576388	0.023	
H25	0.343924	0.555144	−0.576906	0.023	
N21	0.181957	0.368664	−0.184415	0.018	
H26	0.222515	0.237590	−0.134797	0.023	
H27	0.226674	0.524821	−0.163233	0.023	
H28	0.178843	0.326892	−0.253256	0.023	
H29	0.104417	0.394772	−0.184320	0.023	

### (II_DFT) Hydrogen-bond geometry (Å, º)

**Table:**

*D*—H···*A*	*D*—H	H···*A*	*D*···*A*	*D*—H···*A*
O17—H18···O11	0.99	1.75	2.630	146
N20—H22···O13^i^	1.04	1.76	2.798	177
N20—H23···O16^ii^	0.95	1.90	2.755	148
N20—H24···O14^i^	1.03	1.82	2.831	170
N20—H25···O15^iii^	1.03	1.82	2.831	167
N21—H26···O11	1.04	1.75	2.767	164
N21—H27···O12^iv^	1.04	1.69	2.730	176
N21—H28···O12^iii^	1.03	1.78	2.798	168
N21—H29···O14^v^	1.02	2.13	2.996	141

Symmetry codes: (i) *x*, *y*+1, *z*−1; (ii) −*x*+1/2, *y*+3/2, −*z*−1/2; (iii) −*x*+1/2, *y*+1/2, −*z*−1/2; (iv) *x*, *y*+1, *z*; (v) *x*−1/2, −*y*+1/2, *z*−1/2.
